# Moving through migrant psychiatry: asylum seeking in Europe, forced mobility, and anthropology as interdisciplinary intervention

**DOI:** 10.3389/fpsyt.2025.1596290

**Published:** 2025-07-07

**Authors:** Nichola Khan

**Affiliations:** Research Institute of Geography and the Lived Environment, University of Edinburgh, Edinburgh, United Kingdom

**Keywords:** migrant psychiatry, asylum seekers, forced mobility, anthropology, wandering, Paris (France)

## Abstract

This perspective reflects on the relationship between migrant psychiatry and asylum seeking in Europe, drawing on anthropological fieldwork in a public migrant psychiatry clinic and mobile psychiatry teams serving asylum seekers, refused asylum seekers, and homeless migrants in France. Restrictive EU migration policies have produced protracted forms of “wandering” that may last for years; a sedentarist emphasis in national migrant services has generally not kept pace. Calls by international agencies to protect the mental health of refugees and displaced people are conflicting with a hostile policy backlash by national governments, delimiting a contradictory situation. This perspective discusses ways movements of migrants across countries and discontinuous and uneven healthcare and asylum infrastructures are shaping clinical expressions of illness and intervention and the asylum clinic as a critical site of inquiry. It develops on anthropology as an interdisciplinary intervention that can more roundly align ways in which migrant patients, clinical services, and professionals move across sectoral boundaries, account for contested political fields and multiple registers of interpretation, and answer some questions arising at their juncture.

## Introduction

By the end of 2023, about 117.3 million people worldwide were forcibly displaced due to persecution, armed conflict, human rights violations, and increasing climate change impacts (1). In Europe, a hardening of border policy and intensification of protection elsewhere and internal protection practices have deterred refugee arrivals through removal to “third country” policies and containment in “safe havens” (2). Recent policy movements in national governments echo rising populism and anti-migrant rhetoric, such as the “stop the boats” mantra in Britain. This is creating a repressive policy backlash, which is in opposition to calls by international organizations, for example, the Platform for International Cooperation on Undocumented Migrants (3), which highlights the need for inclusive policies that grant access to undocumented people to public services and pathways out of irregularity. The International Organization for Migration likewise has a mandate to protect and promote the mental health of migrants, including displaced people, refugees, and asylum seekers—and advocates an interdisciplinary, flexible approach. These framings are positioning migrant psychiatry as a specialist field that aligns global health priorities with state funding, national healthcare, and the rationale of a “treatment gap,” such as launched the field of Global Mental Health (4), expanded for the Global North. Interdisciplinary approaches are important in accounting for the impacts of displacement, forms of forced mobility, and a contradictory, hostile policy field on migrant mental health and services.

Migrant psychiatry in Europe is a dynamic and potential site of mobile conjunction and historical-political significance between asylum seeking, psychiatric services across diverse sectors, chronic displacement, and exclusions involving refugees from different countries. While the specialism is expanding, a sedentarist emphasis in public services has generally not kept pace. Forced mobility related to refugee experiences interacting with exclusion and temporariness in Europe lies at the heart of migrant mental illness and its adjudication, but this aspect has been less comprehensively studied. Research tends to categorize pre-migration experiences of war, persecution, and torture; migration itself; and post-migration adjustment processes such as acculturation in the therapeutic management of patients (5). Interdisciplinary perspectives can mitigate the focus on the individual as the locus of intervention and the tendency to downplay the political “disorder” of administrative wandering, legal and social precarity, hostile policy environments, and structural racism. Research into political and social conditions affecting migrant mental illness in Europe is generally lacking. France is one exception, where widespread migrant homelessness, exclusion, asylum refusals, and struggles for legal recognition are producing a richly textured corpus of knowledge that has informed interventions in migrant mental health (6).

This perspective develops on the case of migrant psychiatry in France. It draws from anthropological fieldwork since 2023 in a public migrant psychiatry hospital outpatient clinic and in mobile psychiatry teams across Paris—in temporary and emergency shelters and daycentres serving asylum seekers, refused asylum seekers, and homeless migrants. It involves almost eighty observations of psychiatric consultations, interviews, and patient focus groups conducted with interpreters. It is informed by hospital-based research since 2017 on migrant health in greater Paris (7) and longer term research on refugee migration across Asia and Europe (8). It builds on relationships formed during an International Fellowship held at the Institut Convergences Migrations (2020-23) and with clinical practitioners and services across the Groupe Hospitalier Universitaire Paris Psychiatrie & Neuroscience (GHU Paris). It argues for a perspective on ways the multiply configured movements of migrants through sites of medical and asylum infrastructure and the city are shaping both clinical expressions of illness and intervention. This is germane to Solomon (9), for whom “‘trauma medicine is a process of traffic’…that exemplifies clinical kinetics … and a problem of how to move … as much as what to know.” The fieldwork has highlighted ways that therapeutics are adapting, and must adapt, to risks to mental health arising from the political order. This bears on the asylum clinic as a site of political as well as medical inquiry. It implicates anthropology as a kind of interdisciplinary “intervention” that can analyze interactions of public psychiatry and a longer trajectory of migrant mobility in migration, disrupt boundaries of clinical and political orthodoxy, and innovate practice around migrant mental health in complex urban environments.

## Forced mobility and wandering in France

In January 2025, there were 2.048 million residents in Paris and 12 million in the greater Paris region. The French national agency OFPRA (Office for the Protection of Refugees and Stateless Persons National Agency) reported 167,432 pending asylum applications in 2023 and almost 52,000 new applications in the first four months of 2024, with applicants originating primarily from Afghanistan, Syria, Turkey, Congo, Guinea, Ivory Coast, and Bangladesh (10). French psychiatry encompasses a complex, disaggregated terrain in which asylum seekers and rejected asylum seekers are largely excluded from social and healthcare systems (11). Although French healthcare provides universal coverage for all, the health insurance system and “sectorization” of services disadvantage those without a fixed address. Restrictive migration policies have produced protracted forms of administrative “wandering” (12), which may last for years. Under EU-Dublin procedures, which process asylum seekers in the European country of first arrival, according to fingerprints recorded in EURODAC files, those refused asylum typically move countries and submit new applications elsewhere.

Once OFPRA registers an asylum application, appellants become “refugees/beneficiaries of subsidiary protection” and receive temporary accommodation while awaiting an interview. Their healthcare is administered (sectorized) in that district. Once refused or entering Dublin procedures, they lose accommodation and social assistance, although they may appeal. The appeal period against deportation is highly stressful. Many patients arrive in clinics at this time. Petitions may be multiple, and applicants are subject to house arrest or immediate deportation. Some make repeated appeals, are deported, return, and reapply. Exiting Dublin procedures may take months or years. Success only grants petitioners the right to have applications heard in France.

Understanding French urban environments and their restrictions also registers the claims of (predominantly Muslim) migrants, stateless people, asylum seekers, and refugees as the Arendtian “right to have rights” (13). This implicates transhistorical frontiers and relations of Europe to its former colonies; coloniality in migrant camps, anti-Muslim racism, deportations, the deaths of 33.7k migrants between 2001 and 20–17 in the “Black Mediterranean” (14)—and longstanding marginalizations of diasporic Arabs, Africans, and asylum seekers in ghettoized Parisian suburbs. It signifies migration with the subjective search for place and implicates the refusal of the foreigner, beyond the administrative refusal, in ‘wandering’ as a political symptom (15). It problematizes the interface of asylum law and psychiatric medicine in ways people are officially recognized or interpellated primarily as sufferers of trauma and petitioners of the state, and their experiences are deprioritized in that process.

Regarding the situation in Paris, there are around 50,000 asylum seekers overall, according to City Hall, with accommodation provided for around 30,000. These figures exclude rough sleepers, those requalifying from previous years, or those under the EU-Dublin procedures. Asylum seekers in Paris may be housed in emergency accommodation centers; many refused applicants become street homeless. Living conditions are punctuated by voluntary and institutional structures, varying definitions of reception and exclusion, and constant movements between homelessness and nonlinear accommodation (16). Estimates, hard to verify, number between 400,000 and 600,000 undocumented migrants across France, mostly concentrated in greater Paris. Many have no access to basic subsistence requirements of food, housing, and healthcare—and live in conditions the European Court of Human Rights (17) condemns as inhuman and degrading. While most migrants in homeless camps in Paris are under Dublin procedures, on the run, refused, or undocumented, the homelessness of recognized refugees is also increasing. Overland journeys to France can take three to five years; asylum applications take more years to process, with 25%–30% successful first time. Those granted refugee status may claim permanent residence after ten years. The dismantling of camps and dispersals by the police outside the city occur alongside continuous return movements to Paris. Analyses in turn pivot between paradigms of “repression” and “assistance,” and questions regarding whether migration and homelessness should be treated as separate administrative and legal distinctions (18). Medical research typically follows policy, and research with non-Francophone homeless migrants is lacking (6). While insights can be gained from mental health research in inner cities with homeless populations, migrant homelessness has a different profile. It is linked to difficulties related to asylum seeking and administrative status, language barriers and exclusion from citizenship, employment rights, and the rental sector—rather than lifetime traumatic events and mental health problems, addiction, crime, or imprisonment characterizing the homelessness of French-born affectees (19). Homeless migrants also include more women and families with children: while affectees are generally younger and healthier, they experience situations of greater isolation, extreme destitution, and societal racism and stigma (ibid).

## The French asylum clinic

Migrants’ distress in Paris is not straightforwardly accommodated in services wherein care is discontinuous, unevenly dispersed, and inadequate for people who are psychologically exhausted after years on the move (20). Mental healthcare services for migrants are split between independent medical centres, state-funded public health services, and diverse non-governmental organizations operating under the public health umbrella SAMU (Service d’Aide Médicale Urgente). Public healthcare systems are more accustomed to dealing with chronic and severe problems such as psychotic disorders. This leaves the NGO sector in many instances to assume the burden, despite their lower reception and care capacity. Studies highlight the medical certification of post-traumatic stress disorder (PTSD) as a border technology governing refugee status through the asylum claim in France (21). PTSD is unusual insofar as it positions psychiatric illness as often an applicant’s last appeal to juridical inclusion (22). At the same time, migrants seeking asylum are more likely than other groups to experience worse mental health problems and long-term homelessness. In Europe’s racialized hierarchies of migration, access to mental healthcare is unequal; racism interacting with the visible precarity of migrants in public space means Africans fare worse, being subject to greater state surveillance and control and more likely to receive asylum refusals (23).

The clinic in my field site is the first non-sectorized public psychiatric outpatient hospital service in France. It was established in 2021 by the French regional health agency to address an urgent need identified in psychiatric services for “migrants” living in extreme precarity in greater Paris (Île-de-France). It developed from the mobile psychiatric service created in 2005 to provide frontline psychiatry to people in unstable housing, not migrants specifically. It treats migrants regardless of if or where they are housed. Around half the patients are homeless. To adapt to these conditions, it runs a no-appointment system, and treatment is not time-restricted. This policy is very effective. The service receives referrals from mobile teams, reception and emergency centres, and NGOs. The team includes a social worker, two psychiatrists, and a psychiatric nurse. It employs four full-time interpreter–mediators and is the only service in Île-de-France to do so. The interpreters are former refugees with lived experience of the conditions facing patients. They work through clinical, housing, and social checklists and accompany patients to OFPRA hearings. They mediate the social and administrative environment and may share their experiences. Patients stabilize largely in relation to the progress of asylum applications, highlighting political risk factors arising at the juncture of asylum policy and social conditions, housing particularly.

At a clinical level, cases of asylum seekers—as in this field site—commonly involve cumulative trauma events, co-morbidities between post-traumatic stress disorder (PTSD) and depressive and anxiety disorders (24), with a seesaw evolution due to sudden linked losses of accommodation and rights, protracted asylum applications, and repeated refusals. Guidelines developed for specific disorders, notably PTSD, typically do not account for co-morbidities and psychological distress. Language barriers, a low use of interpreters in France, and long-term mobility all significantly impact migrants’ ability to access healthcare and the treatment options available (25). In terms of PTSD especially, first-line treatment proposals tend to prioritize psychological over pharmacological treatments, with sometimes severe consequences. Implementing psychological treatment techniques requires in-depth interpreting, specialized training and supervision, regular appointments, and proximity to appointments—which are largely incompatible with the forced mobility of migrant populations. Pharmacological treatment requires fewer appointments and has greater predictable effectiveness. By exceeding institutional norms in public psychiatry and also filling gaps in the landscape of asylum governance, the clinic is building ethical orientation and its own culture of professional practice. It addresses mental health needs in tandem with social support with housing and asylum applications. Its practices are pragmatic and continually adjusted as migrants move through the city and asylum process.

## Discussion and implications: anthropology as a mode of intervention

This perspective underlines the role of anthropology as a mode of “intervention” from the vantage of mobility, incorporating migrant and practitioner perspectives. It proposes intervention as an ethical mode of interdisciplinary inquiry with practical consequence. Ethical questions pertain to ways anthropologists can help to build care systems around the lives of those treated and problematize power relations in ways migrant traumas are used as a mandate for intervention by international organizations or in political agendas (26). This implicates the evolution of clinical need, ways concepts such as need or “gap” work in regard to political pressures, therapeutics, and social categorizations (27); and tensions around migrant psychiatry as an expanding medical endeavor. As Das (28) proposes, we discover limits of a concept in the ways it moves, in “what might be thought of as roaming in the space of possibilities”.

Anthropologists are a valuable resource for medical professionals dealing with increasing global hyperdiversity, especially in large European cities. They have contributed revisions to psychiatric nosology by drawing on anthropology’s classic traditions of ethnography and long-term fieldwork and a rich corpus of disciplinary knowledge on mental illness and cultural variations (29). A key objective is to disentangle the structural from the political without losing sight of anthropology’s traditional purview on culture—and to incorporate ethnographic and clinical insights from research with specific communities and long-term fieldwork in different locations, such as with Afghan refugees (8), and from fieldwork outside the clinic.

Anthropologists can also reveal diverse encounters of medical knowledge and power in ways the categories of “migrant,” “asylum seeker,” and “trauma” operate in the governance of war, state borders, international law, and humanitarianism. Criticisms have addressed ways PTSD tends to prioritize war-related trauma at the expense of ways suffering is produced within complex dynamics of racial capitalist regimes propelling migration. For clinicians who must work pragmatically to help patients within public health systems, regardless of their political positions, this can offer a valuable critical position. For refugees in Europe who endure multiple losses and stressors related to migration, trauma may not be the only difficulty they experience, but one of many producing vicious cycles that can transform transient symptoms into severe disorders. These are patently tied to asylum refusals, homelessness, and exclusion. Analyzing the interplay of individual, social, and political “disorders” is a core concern of psychiatric anthropology.

Further implications from the example in Paris are relevant to ways migrant psychiatry is developing as a distinctive social mode of practice and an ethical striving toward structurally different ways of working. The role of structural violence in conditioning the social distribution of mental illness demands critical scrutiny. The clinic represents an urban administrative unit that is also a transformative space and a new node of public psychiatry within a broader grid of mental health infrastructure in which the public hospital and its multiple parts are all moved unequally. Its non-appointment, non-time-restricted system of treatment is crucial for ensuring that care, including follow-up support, can accommodate patients with highly mobile, unstable lives. This has greater effectiveness with regard to ensuring treatment adherence and correct dosing. It means clinicians can immediately respond to sudden losses of accommodation by changing pharmacological treatments, for example, to suit sleeping on the street. Working with an onsite social worker can simultaneously assist patients with alternative housing support and asylum appeals. By incorporating interpreter-mediators with lived experiences as refugees, the service builds more authentically around the social and cultural lives of migrants, including language barriers, and especially where their experiences are selectively prioritized, ignored, or misrepresented. Interpreter-mediators not only acculturate patients to the complex administrative asylum process but triangulate cultural knowledge with clinicians to adapt pharmacological treatments for Muslim patients, for example, during Ramadan, who may be fasting during daylight hours. This ensures services remain relevant to the lived realities and humanity of migrant life and that migrants feel less alienated by therapeutic processes. Future research directions implicate other situations with refugees and displaced people where doctors must provide for urgently shifting social needs; in inner cities where psychiatric care requires daily adjusting to keep the patient’s life situation from deteriorating; and clinical practice must adapt to everyday urgencies over abstract principles. By underlining ways migrant psychiatry in Paris works with severe marginalization and multiple barriers to healthcare, this clinic is improving understandings of migrant patients’ experiences, contributing to debates about policy and practice in this site and in mobile outreach services, and enriching theoretical understandings of medicine as a critical site of the urban. It underscores the need for clinical services in migrant psychiatry to “move” more responsively with migrants and across the range of medical, social, and legal decision-making services.

## Data Availability

The original contributions presented in the study are included in the article. Further inquiries can be directed to the corresponding author.
